# The Use of the Symani Surgical System® in Emergency Hand Trauma Care

**DOI:** 10.1177/15533506241262568

**Published:** 2024-06-17

**Authors:** Nadjib Dastagir, Doha Obed, Martynas Tamulevicius, Khaled Dastagir, Peter Maria Vogt

**Affiliations:** 1 Department of Plastic, Aesthetic, Hand and Reconstructive Surgery, 9177 Hannover Medical School, Hannover, Germany

**Keywords:** robotic surgery, plastic surgery, surgical education

## Abstract

**Background:** The use of robotic systems for microsurgery has gained popularity in recent years. Despite its drawbacks, such as increased learning time and lack of haptic feedback, robot-assisted microsurgery is beneficial for emergency care due to its reduced risk of tremor and fatigue. The Symani Surgical System® is 1 example of this advanced technology. The device offers a range of possibilities in the field of microsurgery by combining precision and dexterity, revolutionizing microsurgical procedures. This article explores the applications of the Symani in microsurgical procedures in emergency hand trauma care, highlighting its advantages and limitations. **Material and Methods:** We present the results of 62 anastomoses of blood vessels under .8 mm diameter after hand trauma. 31 anastomoses were conducted using the Symani Surgical System®, and the other 31 were done as a control group in hand-sewn technique. **Study Sample:** The patient characteristics, including sex, age, and risk factors, were matched. Results: We found no significant differences in the anastomosis surgery length when performed with the Symani (arterial 17.3 ± 1.9 min; venous 11.5 ± 1.3 min) vs the hand-sewn technique (arterial 16.1 ± 1.4 min; venous 10.2 ± 1.8 min). Additionally, the learning curve consistently decreased over time, with the 10th surgery taking 30% (arterial) less time. **Conclusion:** Our study indicates that robot-assisted microsurgery can help surgeons maintain a relaxed and focused state while producing results comparable to hand-sutured procedures in emergency care.

## Introduction

Supermicrosurgery, a highly delicate procedure that involves the anastomosis of vessels less than .8 mm in diameter, has revolutionized surgical procedures in the plastic and reconstructive field.^1^ It requires precision and attention to detail, as even the slightest hand tremor or fatigue can have significant consequences.^2,3^ Traumatic injuries to the hand and fingers are common in emergency medicine and require advanced microsurgical methods to reconstruct the nerves and vasculature, minimizing functional losses.^4^ In emergency care and microsurgery, maintaining focus and concentration is crucial to ensure that every patient receives the same level of treatment, regardless of their arrival time at the hospital. Previous studies showed that the Symani Surgical System® may be advantageous due to its reduced risk of tremors and protection from fatigue.^5^ This allows for sustained concentration over extended periods of time. The robotic system reduces the scale of hand motions performed by the surgeon by 7 to 20 times.^6^ Moreover, a comparison study by Willems et al. found that the use of robotic microsurgery systems was superior over manual surgery in technically difficult exposures by reducing surgery time and improving the surgeon’s comfort rating.^7^ The goal of incorporating robotic assistance is not only to achieve surgeries that were previously beyond the scope of manual repair, such as distal finger replantation, but also to help the surgeon maintain concentration and focus for upcoming surgeries during the day.

Initial attempts applying robotic surgical systems in microsurgery were performed using the Da Vinci®, which was originally designed for laparoscopic procedures. While this system offered advantages such as wristed instruments and tremor reduction, it was constrained by limitations in visual magnification, instrument size, and motion scaling, which are essential for microsurgery.^8^ The MUSA® system, developed by MicroSure, represents an advancement in the field of microsurgery. The device is designed for integration into conventional surgical environments and is compatible with existing microsurgical instruments, thereby offering the option of more versatile attachments and decreased equipment costs.^9^ In contrast, the Symani system is distinguished by the use of NanoWrist® instruments. These instruments permit movements that closely resemble those of the human hand and offer superior ergonomics, precision, and versatility in comparison to MUSA®.^10-12^ To integrate these methodologies, it is necessary for surgeons to gain experience in robotic-assistance surgeries. Lack of experience using these systems and learning time can greatly influence the operation time and potentially affect the surgical outcome.^6^ It is therefore crucial to compare the results of pre-established microsurgical methods to robotic-assistance methods in standard surgeries. Current literature shows that surgical times increase when microsurgeons use robotic systems, however as more surgical attempts are made this time decreases.^13,14^

In this study, we aimed to show that it is possible to perform microsurgeries with the Symani Surgical System® in emergency hand trauma care with comparable times to the hand-sewing technique, and the learning curve improves with increased experience working with the device. Additionally, we have demonstrated that the Symani Surgical System® can provide advantages in microsurgery due to its flexible robotic arm and improved ergonomics, which can prevent fatigue and increase precision. Our results suggest that (super)microsurgical techniques could be incorporated into emergency hand trauma care procedures in the future, once the learning curve is overcome, and can assist surgeons in maintaining focus for upcoming surgeries.

## Material and Methods

The patients included in this study were admitted to the emergency care at our institute between 2022 to 2024. Cases were consecutive. Eligibility criteria included being at least 18 years old, having a good understanding of the German language, and having sufficient tissue available for primary reconstruction of the injuries. The study was performed in accordance with the ethical standards of the Declaration of Helsinki and all procedures were performed in compliance with relevant laws and institutional guidelines. We conducted a retrospective study comparing the outcomes of a total of 21 patients requiring blood vessel anastomosis after finger injury at our clinic. 10 patients received Symani-assisted arterial (n = 15), venous (n = 12) and arteriovenous (n = 4) anastomoses. 11 patients received hand-sewn arterial (n = 18), venous (n = 11), and arteriovenous (n = 2) anastomoses. The Symani Surgical System® (Medical Microinstruments, MMI, Calci, Italy) was used for all robotic-assisted anastomoses. A standard surgical microscope was used for all procedures in this study. All of the anastomoses were sutured in the interrupted technique. The motion scaling of for the Symani system was set to 10x. An assistant surgeon was seated at the surgical site operating manually. All surgeries included in the study were performed by the author Dastagir K., an experienced microsurgeon. Data were analyzed using GraphPad Prism Version 9.2.0 for descriptive statistics, unpaired *t* test, and ANOVA. Results were considered significant if *P* < .05.

## Results

We compared the anastomosis times between a group of 10 patients receiving Symani-assisted sewing and 11 patients receiving hand-sewing. Patients were matched for sex, age, risk factors, and American Society of Anesthesiology (ASA) score. The median age for patients treated with the Symani was 40 (±4.4), and patients treated by hand-sewing was 43 (±5.9) (Table 1). The type of surgeries varied between the following: replantation (n = 8), finger blood vessel injury (n = 13). All patients received treatment in the hand after trauma (n = 21). In total, 31 Symani-assisted anastomoses were performed. Of these, anastomosis type varied between: arterial (n = 15), venous (n = 12), arteriovenous (n = 4) (venous interposition for arterial reconstruction) (Table 2). No postoperative complications related to the anastomoses were observed in cases performed using the Symani Surgical System ®.

**Table 1.** Patient characteristics.

Groups	SymaNI	Manually
Patient characteristics		
Number of patients (N)	10	11
Age (years, mean ± SEM)	40 (±4.4)	43 (±5.9)
ASA classifcation, (n)		
I	7	6
II	3	4
III		1
Type of surgery (N)		
Finger replantation	4	4
Finger injuries	15	14

**Table 2.** Anastomosis type.

Anastomosis type	Symani (N)	Manually (N)
Arterial	15	18
Venous	12	11
Arteriovenous (venous interposition)	4	2

**Figure 1.**
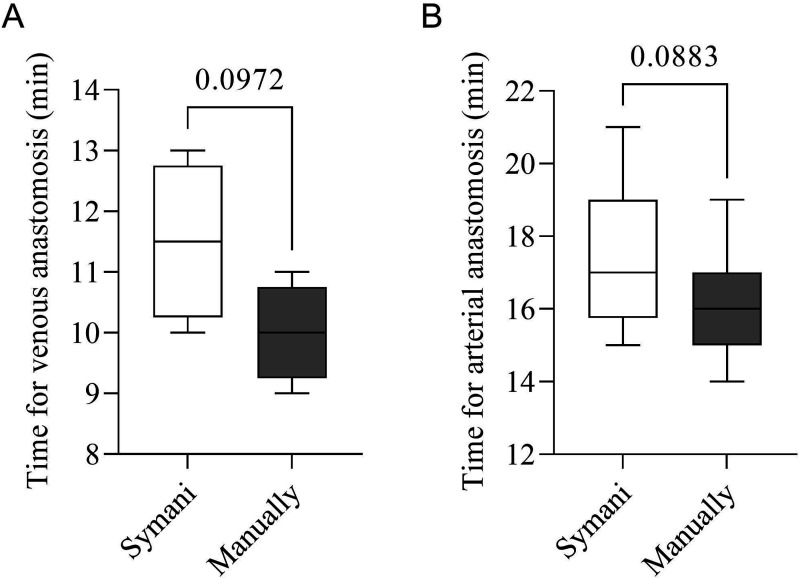
Time for Anastomosis Symani vs manual (A) venous (B) arterial.

**Figure 2.**
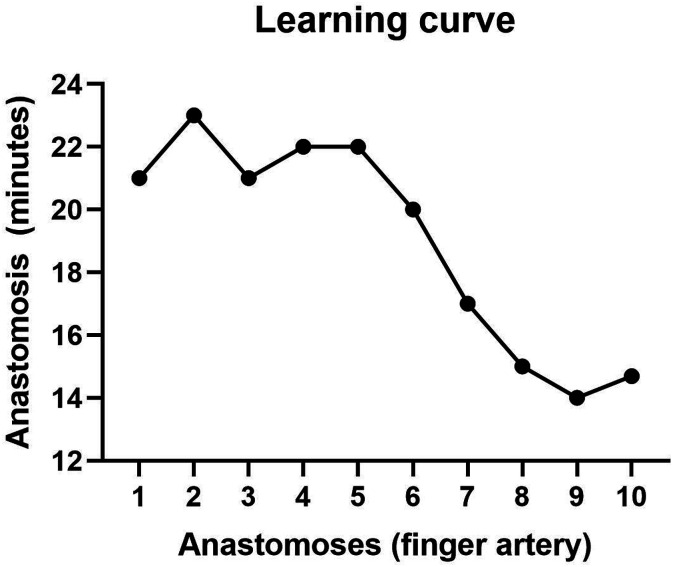
Learning curve.

**Figure 3.**
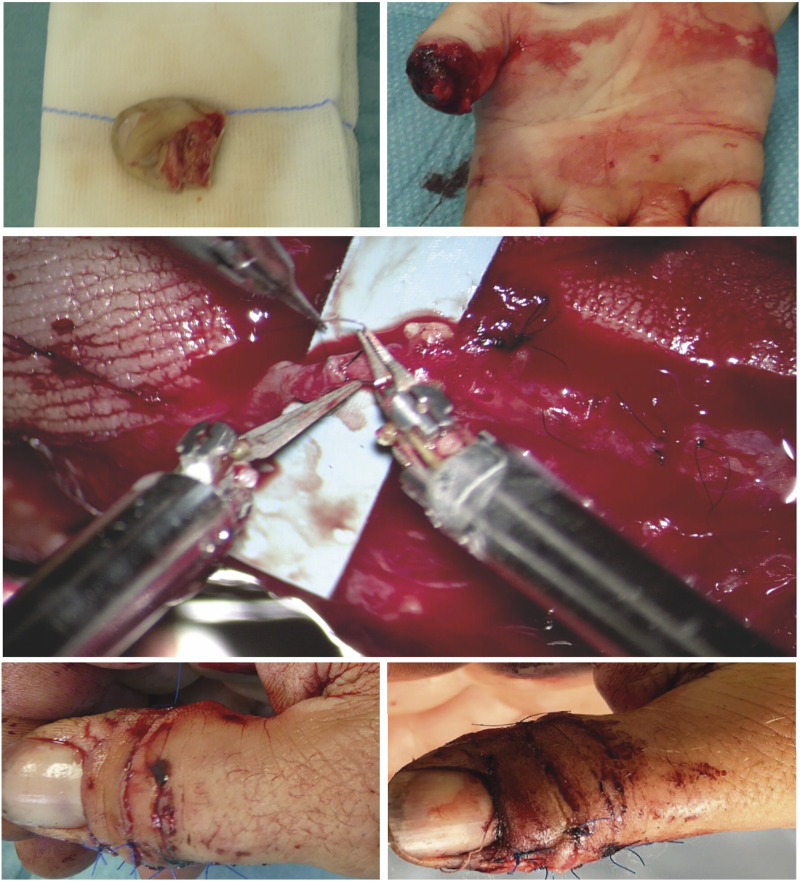
Thumb replantation.

We found no significant differences in the anastomosis surgery length when performed with the Symani (arterial 17.3 ± 1.9 min), (venous 11.5 ± 1.3 min) vs the hand-sewn technique (arterial 16.1 ± 1.4 min) *P* = .0883, (venous 10.2 ± 1.8 min) *P* = .0972 (Figure 1). Patency was tested using milking test, indocyanine green (ICG), and clinical evaluation. A total of 100% of anastomoses were patented. A learning curve was calculated from the results of 10 finger arteria reconstruction using the Symani Surgical System® (Figure 2). The mean anastomosis time readily decreased with more surgeries (Figure 3). The first Symani-assisted surgery required 21 minutes, by the 10^th^ surgery this was reduced to 14.7 minutes (30%). The average anastomosis time of the first 5 surgeries with the Symani was 22 minutes, compared to 16 minutes for the last 5 surgeries, demonstrating a significant reduction of anastomosis time (*P* = .04). The average hand-sewn anastomoses performed by our microsurgeon were approximately 16.2 minutes (arterial) and 10.1 minutes (venous), demonstrating that the Symani-assisted technique came close to reaching this threshold. Due to the smaller size of the veins and the reduced need for sutures, anastomosis times of the arteries are shorter.

Despite requiring a learning curve, the surgeon reported improved ergonomics leading to increased concentration when using the Symani Surgical System®. The steadily decreasing surgical time suggests that with further training the anastomosis time will be highly similar to hand-sewn surgery. The overall surgery time for the Symani-assisted surgeries was 18 minutes longer due to machine setup, however this was reduced to 15 minutes by the 10^th^ surgery as the staff became more familiar with the equipment. With the machine’s integration into routine microsurgery operations, it is anticipated this time will also decrease as staff have more experience with it.

## Discussion

Symani Surgical System® microsurgery devices have been on the market for more than 5 years.^15^ The use of robotics is an innovation in the field of microsurgery, however it has not been integrated in many surgical units.^14,16^ This is largely due to the high cost of the equipment, training expenses, and the steep learning curve.^17^ This study demonstrates the feasibility of utilizing the Symani Surgical System® for emergency hand trauma care procedures. We compared 11 matched patients receiving manual sewing with 10 patients receiving Symani-assisted blood vessel anastomoses after hand trauma. The surgery types included finger replantation and finger blood vessel injury (Table 1). Our results demonstrate that surgical time with the Symani greatly decreased as our microsurgeon performed more anastomoses (Figure 2). Furthermore, there was no significant difference in surgical time between hand-sewing and Symani-assisted sewing, showing it can be successfully integrated into routine surgical procedures (Figure 1). A recent meta-analysis by Susanto et al. found that robot-assisted microvascular anastomosis time was longer than manual anastomosis, in some cases requiring twice the length of time.^13^ Differences in mean anastomosis time are likely to vary due to differences in the operating microsurgeon’s experience level, the operative procedure, and the technical difficulty of the operation. As more clinics report on the results of robotic-assisted microsurgery cases it will aid in identifying trends in anastomosis time.

The advantages of robotic surgery systems are already documented in the literature, such as increased precision and accuracy, and their ability to circumvent the psychological and physical limits of the surgeon such as tremor.^8^ Our surgeon reported improved concentration and reduced fatigue between operations, which is necessary for providing effective treatment to patients in emergency care. Coordination of the feet and hands is crucial for achieving precise results when operating the pedal and Symani surgical arms simultaneously. The use of an exoscope in conjunction with the Symani system may provide additional ergonomic benefits, which could be explored in future studies. While additional studies are necessary to determine other surgeons’ experiences with the equipment, this could lead to reduced operation time in emergency care and, more importantly, greater surgical efficiency:

It is important to consider that robot-assisted devices require a surgical team to establish the system in the operating room. This equipment setup has its own learning curve, which can lead to increased surgical times when the team is less familiar with the device. The preparation of the Symani system requires the involvement of 2 surgical technical assistants, who are responsible for draping the robotic system and the surgeon’s chair, as well as the manipulators. It is of paramount importance to ensure that the system remains sterile throughout the procedure, which can be an intricate and time-consuming process. The necessity for meticulous draping to maintain sterility adds to the complexity of the setup. The positioning of the Symani operating unit in close proximity to the operating table, while ensuring sufficient space for an assistant surgeon and surgical nurse, represents a significant challenge. This setup necessitates precise placement to avoid interference and ensure smooth operation. The arms must be positioned in a manner that allows for unrestricted and collision-free movements, which can be difficult to achieve, particularly in a crowded operating room environment. We found that after 10 anastomosis surgeries, this equipment setup time improved. As the use of the Symani system becomes more routine for both the staff and microsurgeons it can be expected this time will lessen.

Adopting the Symani Surgical System® requires a considerable investment in training and certification for clinical use. Intensive preclinical training sessions using vessel models are essential to simulate intraoperative conditions and develop proficiency with the robotic system. This comprehensive training curriculum demands significant time and financial resources from surgical teams.^18^ Furthermore, the high initial capital cost of acquiring the Symani system itself presents a financial challenge, especially for smaller health care facilities with limited budgets. Jimenez et al. reported in 2022 that out of 184 plastic surgery residents 73% expressed that the cost was the most notable barrier to widespread robotic implementation.^19^ Ongoing expenses for consumables and maintenance further contribute to the long-term operational costs, highlighting the importance of a thorough cost-benefit analysis before implementing this advanced robotic technology.^20^ Shakir et al. reported that the cost of robotic-assisted collection of deep inferior epigastric perforator (DIEP) flap tissue cost $1500, 3 and 6 fold times more expensive than total extraperitoneal laproscopic harvest and endoscopic harvest.^21^ Additionally, Gundlapalli et al. reported higher costs associated with robotic-assisted surgery, however they noted the total patient charge was comparable, with a robot-assisted breast reconstruction using a DIEP flap costing $16,300, compared to the standard DIEP flap reconstruction costing $14,800.^22^ Increased reporting on the costs associated with robot-assisted surgery compared to manual techniques would allow for improved economical cost-benefit analysis associated with incorporating robotic surgical systems.

Despite these financial hurdles, the Symani Surgical System® received FDA approval in April 2024 for use in the US, following its CE marking in 2019, which allowed commercial use in Europe and parts of Asia Pacific.^23^ The system has been used in nearly 1000 clinical cases in Europe, and its integration into clinical use is expected to increase with the recent FDA clearance.^24^

The results of the study must be interpreted considering the following limitations. Our study is a monocenter study, and all of the surgeries were performed by 1 experienced microsurgeon. It would be ideal to compare learning curves across multiple clinics to determine the range of learning time and identify possible factors contributing to improved robotic integration.

The use of robotic systems in emergency care can protect surgeons from fatigue and provide them with the opportunity to perform surgeries with the same level of focus. Furthermore, it is a new frontier that will allow for advanced techniques that are currently not possible due to human limits. By enabling anastomosis of smaller vessels, it will be possible to perform techniques such as distal finger replantation. Moreover, this technology elevates current surgical standards by enabling greater accuracy and precision when working with small vessels, thereby reducing patient risk and enhancing healing outcomes. As more health care systems adopt this technology, the accumulation of data will provide a deeper understanding of its optimal applications, strengths, and limitations.

### Conclusion

The Symani Surgical System® was found to be feasible for emergency hand trauma care, performing microsurgical anastomoses with times comparable to traditional hand-sewn techniques after overcoming the initial learning curve. The study observed a significant reduction in surgery time with increased experience, indicating a steep learning curve that improves with practice. The system’s ergonomic design and tremor filtration capabilities helped reduce surgeon fatigue and improve precision, which are critical for maintaining concentration during prolonged surgeries. Future research should focus on multi-center studies to validate these findings across different clinical settings and to explore the long-term benefits and cost-effectiveness of robotic-assisted microsurgery.
